# Correction: Park et al. Defining and Addressing Research Priorities in Cancer Cachexia through Transdisciplinary Collaboration. *Cancers* 2024, *16*, 2364

**DOI:** 10.3390/cancers17060971

**Published:** 2025-03-13

**Authors:** Margaret A. Park, Christopher J. Whelan, Sabeen Ahmed, Tabitha Boeringer, Joel Brown, Tiffany L. Carson, Sylvia L. Crowder, Kenneth Gage, Christopher Gregg, Daniel K. Jeong, Heather S. L. Jim, Andrew R. Judge, Tina M. Mason, Nathan Parker, Smitha Pillai, Aliya Qayyum, Sahana Rajasekhara, Ghulam Rasool, Sara M. Tinsley, Matthew B. Schabath, Paul Stewart, Jeffrey West, Patricia McDonald, Jennifer B. Permuth

**Affiliations:** 1 Department of Gastrointestinal Oncology, H. Lee Moffitt Cancer Center & Research Institute, Tampa, FL 33612, USA; margaret.park@moffitt.org; 2 Department of Biostatistics and Bioinformatics, H. Lee Moffitt Cancer Center & Research Institute, Tampa, FL 33612, USA; paul.stewart@moffitt.org; 3 Department of Metabolism and Cancer Physiology, H. Lee Moffitt Cancer Center & Research Institute, Tampa, FL 33612, USA; virens@darwiniandynamics.org; 4 Department of Machine Learning, H. Lee Moffitt Cancer Center & Research Institute, Tampa, FL 33612, USA; sabeen.ahmed@moffitt.org (S.A.); ghulam.rasool@moffitt.org (G.R.); 5 Department of Drug Discovery, H. Lee Moffitt Cancer Center & Research Institute, Tampa, FL 33612, USA; tabitha.boeringer@moffitt.org (T.B.); smitha.ravindranadhanpillai@moffitt.org (S.P.); 6 Department of Cancer Biology and Evolution, H. Lee Moffitt Cancer Center & Research Institute, Tampa, FL 33612, USA; joel.brown@moffitt.org (J.B.); jeffrey.west@moffitt.org (J.W.); 7 Department of Integrated Mathematical Oncology, H. Lee Moffitt Cancer Center & Research Institute, Tampa, FL 33612, USA; 8 Department of Health Outcomes and Behavior, H. Lee Moffitt Cancer Center & Research Institute, Tampa, FL 33612, USA; tiffany.carson@moffitt.org (T.L.C.); sylvia.crowder@moffitt.org (S.L.C.); heather.jim@moffitt.org (H.S.L.J.); nathan.parker@moffitt.org (N.P.); sara.tinsleyvance@moffitt.org (S.M.T.); 9 Department of Diagnostic Imaging and Interventional Radiology, H. Lee Moffitt Cancer Center & Research Institute, Tampa, FL 33612, USA; kenneth.gage@moffitt.org (K.G.); daniel.jeong@moffitt.org (D.K.J.); aliya.qayyum@moffitt.org (A.Q.); 10 School of Medicine, University of Utah, Salt Lake City, UT 84113, USA; chris@storylinehealth.com; 11 Department of Physical Therapy, University of Florida, Gainesville, FL 32610, USA; arjudge@phhp.ufl.edu; 12 Department of Nursing Research, H. Lee Moffitt Cancer Center & Research Institute, Tampa, FL 33612, USA; tina.mason@moffitt.org; 13 Department of Supportive Care Medicine, H. Lee Moffitt Cancer Center & Research Institute, Tampa, FL 33612, USA; sahana.rajasekhara@moffitt.org; 14 Department of Malignant Hematology, H. Lee Moffitt Cancer Center & Research Institute, Tampa, FL 33612, USA; 15 Department of Cancer Epidemiology, H. Lee Moffitt Cancer Center & Research Institute, Tampa, FL 33612, USA; matthew.schabath@moffitt.org; 16 Lexicon Pharmaceuticals, Inc., Woodlands, TX 77381, USA

## Addition of an Author

Dr. Tiffany L. Carson was inadvertently not included as an author in the original publication [1]. The corrected Author Contributions statement appears here. The authors state that the scientific conclusions are unaffected. This correction was approved by the Academic Editor. The original publication has also been updated.
